# Causes of Puerperal Fever

**Published:** 1870-10

**Authors:** 


### Causes of Puerperal Fever.

At a meeting of the Obstetrical Society in London, in March, this subject came up for discussion (*British Medical Journal*, March 19, 1870):

Dr. Wynn Williams said that the parturient female, if exposed to the influence of the scarlatina poison, might become affected with the disease, which would be modified and rendered more fatal by the peculiar condition of the patient. As to the second class of cases, he was rather skeptical as to their being affected with the scarlet fever itself. During the prevalence of scarlet fever, the atmosphere is not only impregnated with impure air, but also with toxic septic emanations from the putrid throats of the patients. Should these emanations be brought into contact with the discharges of the parturient female, they would act as a kind of ferment. Offensive discharges from the vagina must be treated locally. Dr. Wynn Williams preferred a solution of iodine, as by its volatility, increased by the heat of the body, it was more likely to be brought into contact with any septic poison lurking, it might be, in the folds of the mucous membrane. The same observations would apply to the other classes enumerated by Dr. Hicks—erysipelas, diptheria, etc. Any putrid emanation brought into contact with the discharges of the parturient female would act as a ferment and produce septic poisoning. Dr. Barnes said that puerperal fever was pre-eminently a disease that called for the application of sanitary laws with a view to prevention. This disease still destroyed more lying-in women than any other, probably in England, certainly on the continent, where it killed so many women in lying-in hospitals. The classification of causes pursued by Dr. Hicks resembled that adopted by himself, of dividing puerperal fever into two great classes, the *Autogenetic* and *Heterogenetic*. His own experience coincided with that of Dr. Hicks as to the frequency of of scarlatina among lying-in women. It was propagated by foul linen, by sewage emanations, and by direct connection. Dr. Hicks had noticed the frequency of puerperal fever in new houses. Dr. Barnes' former experience as a medical officer of health enabled him to explain this. Builders dug out the gravel, sold it, and filled in with the foulest putrifiable rubbish.

Where scarlet fever broke out on the second or third day after delivery, he had seen reason to infer that the poison was inoculated at the time of labor. But, in many cases, the mother was subject to the influence of the poison before labor. During pregnancy, especially if she had had scarlatina before, she shared in the power which most had of throwing off the poison. It was only when the excretory organs were once charged with the double work of dealing with the products of gestation and with the poison, that the system broke down, and puerperal fever was produced. The lying-in hospital was a propagating house for every form of puerperal fever. Ehe forms chiefly prevalent in hospitals were scarlatina, erysipelas, and hospital gangrene. Considering the vast predominance of heterogenetic puerperal fever, the question arose whether there existed an essential puerperal fever, arising strictly out of the puerperal state, that could give rise to an epidemic. This he was inclined to doubt. The autogenetic forms proper—those, for example, arising from the decomposition of the retained placenta—did not appear to possess active powers of propagation. But we were rarely in a position in practice to distinguish the contagious from the non-contagious forms. The proofs of contagion were but too common. There were the frequent series of cases in the practice of one man, while neighboring practitioners were free; and he had noticed the sad fact that puerperal fever was usually common among the wives of medical men. Dr. Snow Beck had long been convinced that there was no disease peculiar to the puerperal period that could be called puerperal fever. But there was another class of diseases which were not epidemic and not infectious, and which were most important to be recognized, as that they were very fatal in their effects and yet remarkably amenable to treatment. These arose from the impregnation of the system from offensive or other dlscharges, and were known as septicæmia. This not unfrequently arose from the retention of coagula or portions of placenta within the cavity of the uterus. To admit this, the uterus must be imperfectly contracted; and this condition also allowed the sinuses to remain open, so as to permit absorption of offensive discharges. The treatment consisted in washing out the uterine cavity and all the passages with disinfecting solutions, and giving sulphites internally. A similar practice was equally efficacious in arresting the progress of phlegmasia dolens, which, in the majority of cases, arose from coagulation of blood in the veins, caused by the absorption of offensive discharges from the uterus. When these facts were more generally recognized, the supposed injurious effects of lying-in hospitals, of overcrowding, etc., would cease to have much influence.

